# Nationwide Trends in Osteoporosis in Koreans With Disabilities From 2008 to 2017

**DOI:** 10.1002/jbm4.10747

**Published:** 2023-05-03

**Authors:** Ji Hyoun Kim, So Young Kim, Jong Eun Park, Hyo Jong Kim, Hyun Jeong Jeon, Yeon Yong Kim, Jong‐Hyock Park

**Affiliations:** ^1^ Department of Internal Medicine Chungbuk National University Hospital Cheongju Republic of Korea; ^2^ College of Medicine Chungbuk National University Cheongju Republic of Korea; ^3^ Institute of Health & Science Convergence Chungbuk National University Cheongju Republic of Korea; ^4^ Department of Public Health and Preventive Medicine Chungbuk National University Hospital Cheongju Republic of Korea; ^5^ Department of Rehabilitation Medicine Chungbuk National University Hospital Cheongju Republic of Korea; ^6^ Big Data Steering Department National Health Insurance Service Wonju Republic of Korea; ^7^ Drug Evaluation Department National Institute of Food and Drug Safety Evaluation Cheongju Republic of Korea

**Keywords:** DISABILITY, EPIDEMIOLOGY, KOREA, OSTEOPOROSIS, PREVALENCE

## Abstract

This study examined the 10‐year trends in the prevalence of osteoporosis according to disability grade and type compared with those without disabilities in South Korea. We linked national disability registration data with the National Health Insurance claims data. Age‐ and sex‐standardized prevalence of osteoporosis were analyzed from 2008 to 2017 according to sex, disability type, and disability grade. Adjusted odds ratios for osteoporosis according to disability characteristics in the most recent years' data were also confirmed by multivariate analysis. Over the past decade, the prevalence of osteoporosis has increased in people with disabilities compared with people without disabilities, and the gap has gradually widened from 7% to 15%. By analysis of the most recent year data, both male and female individuals with disabilities had a higher risk of osteoporosis than those without disability (odds ratios [OR] 1.72, 95% confidence interval [CI] 1.70–1.73 in males; OR 1.28, 95% CI 1.27–1.28 in females); the multivariate‐adjusted OR was especially prominent in disability related to respiratory disease (OR 2.07, 95% CI 1.93–2.21 in males; OR 1.74; 95% CI 1.60–1.90 in females), epilepsy (OR 2.16, 95% CI 1.78–2.61 in males; OR 1.71; 95% CI 1.53–1.91 in females), and physical disability types (OR 2.09, 95% CI 2.06–2.21 in males; OR 1.70; 95% CI 1.69–1.71 in females). In conclusion, the prevalence and risk of osteoporosis have increased in people with disabilities in Korea. In particular, the risk of osteoporosis increases significantly in people with respiratory diseases, epilepsy, and physical disability types. © 2023 The Authors. *JBMR Plus* published by Wiley Periodicals LLC on behalf of American Society for Bone and Mineral Research.

## Graphical abstract

Over the past decade, people with disabilities, including males and females, had a higher rate of osteoporosis than those without disabilities, and the gap has gradually widened.

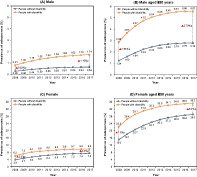

## Introduction

Osteoporosis is a disease defined as decreased bone mass and quality.^(^ ^1^, ^2^ ^)^ Bone fragility and susceptibility to fractures increase with disease progression.^(^ ^1^, ^2^ ^)^ Osteoporosis is also defined by the World Health Organization (WHO) as a bone mineral density (BMD) that is less than 2.5 standard deviations below the mean level for a young adult.^(^ ^3^ ^)^ Several risk factors, including clinical, medical, behavioral, nutritional, and genetic, contribute to the progression of osteoporosis.^(^ ^4^ ^)^ As dominant risk factors for osteoporosis, aging, low body mass index (BMI), and menopause in women are well‐known risk factors for low bone mass and rapid bone loss.^(^ ^5^, ^6^, ^7^, ^8^, ^9^ ^)^ Glucocorticoids are commonly used in the treatment of rheumatic and skeletomuscular diseases. They are representative agents for aggravating BMD.^(^ ^10^ ^)^ Several medical conditions, including gastrointestinal diseases associated with nutrition, hematologic disorders, and hypogonadal states, have been associated with secondary osteoporosis.^(^ ^11^ ^)^

The prevalence of osteoporosis has increased rapidly worldwide in recent decades and now accounts for a large part of the global disease burden.^(^ ^12^, ^13^ ^)^ Asia is reported to have a large proportion of osteoporotic hip fractures.^(^ ^14^ ^)^ The study also reported that this region is expected to account for 37% of all hip fractures by 2025.^(^ ^14^ ^)^ However, Asian studies on osteoporosis are relatively few compared with those in Western countries. A previous Korean study reported that the crude prevalence of osteoporosis in all individuals (40–79 years old) was 13.1% for men and 24.3% for women, satisfying the WHO criteria^(^ ^15^ ^)^ in 2010. Another study reported that the prevalence of osteoporosis in Korea was 7.3% in men and 38.0% in women aged ≥50 years, and the prevalence of osteopenia in Korea was 46.5% in men and 48.7% in women aged ≥50 years in 2014.^(^ ^16^ ^)^

Osteoporosis commonly occurs at the onset of disability, regardless of etiology.^(^ ^17^, ^18^, ^19^ ^)^ However, osteoporosis in people with disabilities has not been well studied. In a previous review of intellectual disabilities, including learning disabilities and intellectual and developmental disability, the association between disability and osteoporosis was surveyed.^(^ ^20^ ^)^ However, no studies could be included in the review; the authors failed to explain this association.^(^ ^20^ ^)^

In particular, it is difficult to prevent and treat osteoporosis and osteoporotic fractures in patients with disabilities because they have low accessibility to medical services owing to environmental and economic reasons. In addition, only few studies have evaluated the association between osteoporosis and disability in the entire population.

This population‐based study aimed to investigate the prevalence and risk of osteoporosis in people with disabilities according to disability severity and type over a 10‐year observation period (2008–2017) using the Korean National Health Insurance Service (NHIS) database.^(^ ^21^ ^)^

## Patients and methods

### Data sources and study population

We linked the national disability registration data with the National Health Information Database (NHID) in South Korea. The NHID is a public database on healthcare utilization, health screening, sociodemographic variables, and death for the entire population of South Korea, maintained by the NHIS.^(^ ^21^ ^)^ From this dataset, we extracted information on the history of inpatient and ambulatory care, drug prescriptions, comorbidities, and sociodemographic variables including age, sex, insurance premium, income level, and residential area.

**Fig. 1:**
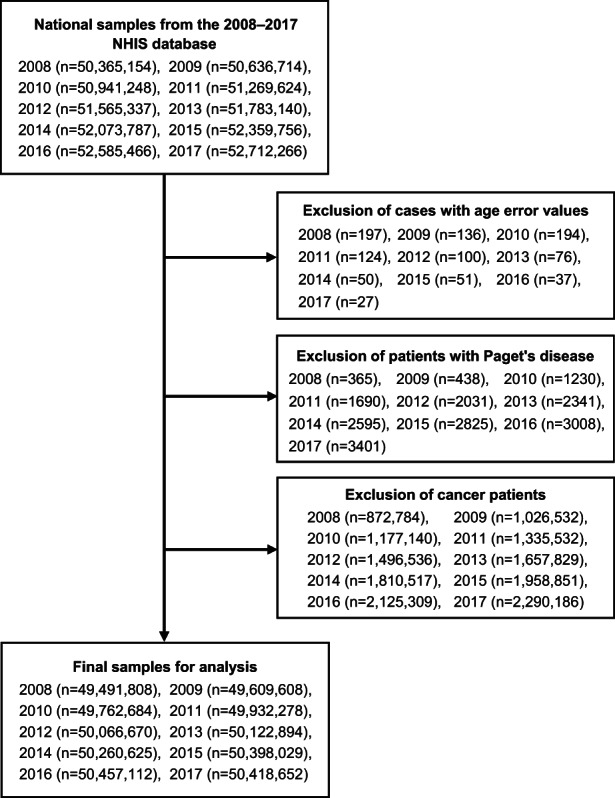
Flowchart of patient selection in the study during 2008–2017.

Using the national disability registration data (NDRD), we collected information disability types and severity levels. The NDRD comprises data from the national registration system for people with disabilities primarily for the provision of welfare benefit.^(^ ^22^ ^)^ Its registration requires submission of appropriate and validated documentation to a local National Pension Service office. The paperwork includes appraised results of disability diagnosis by a specialist physician in the corresponding field according to detailed criteria for the specific disability, as defined by the national disability registration system.^(^ ^23^ ^)^ The database covers 93.8% of the total population with disabilities in 2011.^(^ ^24^ ^)^ Using the Korean personal identification number, disability types and severity levels were linked with the variables selected from the NHID, as mentioned in the previous paragraph. After excluding individuals with a history of Paget's disease and cancer for each year, we analyzed data from more than 50 million individuals throughout the 10‐year period (Fig. 1).

### Study participants and definition of osteoporosis

Osteoporosis was defined under the diagnosis code for osteoporosis with or without pathological fracture (International Classification of Diseases and Related Health Problems, 10th Revision [ICD‐10] M80, M81, M82), which were confirmed if individuals were with prescriptions for osteoporosis or had one or more osteoporotic fractures each year. Medications for osteoporosis included bisphosphonates (alendronate, risedronate, etidronate, clodronate, ibandronate, pamidronate, and zoledronate), selective estrogen receptor modulators (raloxifene and bazedoxifene), synthetic versions of parathyroid hormone (teriparatide), and a monoclonal antibody to the nuclear factor‐κB ligand (denosumab), which are covered by NHIS. Osteoporotic fractures were identified if individuals were diagnosed with vertebral fractures (ICD‐10 M48.4x, M48.5x, S22.0x, S22.1x, S32.0x), hip fractures (ICD‐10 S72.0x, S72.1x), distal radius fractures (ICD‐10 S52.5x, S52.6x), or humerus fractures (ICD‐10 S42.2x, S42.3x) each year. Other variables collected from the NHIS database included age, sex, insurance premium, residential area, and comorbidities. As a proxy measure for actual household income, we used the insurance premium categories of medical aid in quintiles: I (lowest), II, III, and IV (highest), as provided by the NHIS. Insurance premiums were calculated based on income, property, and automobile taxes for each household. Residential areas were grouped into three categories: metropolitan, urban, and rural, based on the Korean ZIP code. Comorbidities were considered potential confounding factors. Comorbidities including diabetes (E10–E14), chronic kidney disease (N18, Z49), and chronic obstructive pulmonary disease (J41–J43), were defined by ICD‐10. The Charlson comorbidity index was used to group study participants into four categories based on the index score: 0, 1–2, 3–4, and ≥5 (the most severe).^(^ ^25^ ^)^ The national disability registration data defines 15 categories of disability. Disability severity is officially graded from 1 (very severe) to 6 (very mild) based on functional losses and clinical impairment as determined by a medical specialist. In this study, disability severity was classified as severe (grades 1–3) or mild (grades 4–6).

### Ethics approval statement

The study protocol was approved by the Institutional Review Board of Chungbuk National University (CBNU‐202010‐HRHR‐0171).

### Statistical analyses

In this serial cross‐sectional study, we calculated the age‐ and sex‐standardized osteoporosis prevalence for each calendar year from 2008 to 2017 according to disability presence and severity by sex and age group. We developed a multivariate logistic regression adjusting for age, income level, residence, comorbidities (diabetes, chronic kidney disease, chronic obstructive pulmonary disease), and Charlson comorbidity index to examine the association between disability and osteoporosis. All analyses were performed using the SAS software version 9·4 (SAS Institute Inc., Cary, NC, USA). Two‐sided *p* values of 0.05 were considered significant.

## Results

### Study participants

The number and baseline characteristics of participants by age group, sex, disability, severity, and type of disability (Table S1) during the period (2008–2017) are summarized in Table 1. In the 2017 analysis, there was no difference in sex distribution compared with 2008, but the proportion of participants aged 50 years or older increased. The proportion of people with severe disabilities decreased from 42.8% to 39.7%, although there was no significant difference in the proportion of people with disabilities in the entire population (Table 1).

**Table 1:** Basic Characteristics of the Study Population by Year (2008–2017)

Characteristic	2008	2009	2010	2011	2012
All participants, *n* (%)	49,491,808	49,609,608	49,762,684	49,932,278	50,066,670
Sex, *n* (%)					
Male	24,915,793 (50.3)	24,976,534 (50.4)	25,064,777 (50.4)	25,163,932 (50.4)	25,238,646 (50.4)
Female	24,576,015 (49.7)	24,633,074 (49.7)	24,697,907 (49.6)	24,768,346 (49.6)	24,828,024 (49.6)
Age (years), mean ± SD	36.3 ± 20.2	36.7 ± 20.3	37.2 ± 20.4	37.6 ± 20.5	38.0 ± 20.6
Age group, *n* (%)					
≤19 years	11,961,489 (24.2)	11,774,719 (23.7)	11,606,699 (23.3)	11,380,161 (22.8)	11,142,554 (22.3)
20–29 years	7,401,534 (15.0)	7,204,053 (14.5)	7,016,039 (14.1)	6,901,962 (13.8)	6,813,972 (13.6)
30–39 years	8,590,408 (17.4)	8,489,212 (17.1)	8,404,669 (16.9)	8,307,459 (16.6)	8,227,447 (16.4)
40–49 years	8,625,872 (17.4)	8,675,563 (17.5)	8,649,877 (17.4)	8,661,468 (17.4)	8,647,540 (17.3)
50–59 years	6,022,884 (12.2)	6,359,463 (12.8)	6,769,950 (13.6)	7,195,468 (14.4)	7,435,128 (14.9)
60–69 years	3,808,063 (7.7)	3,860,101 (7.8)	3,923,718 (7.9)	3,925,802 (7.9)	4,012,698 (8.0)
70–79 years	2,237,904 (4.5)	2,349,829 (4.7)	2,439,109 (4.9)	2,555,607 (5.1)	2,715,031 (5.4)
≥80 years	843,654 (1.7)	896,668 (1.8)	952,623 (1.9)	1,004,351 (2.0)	1,072,300 (2.1)
Status of disability, *n* (%)					
Without disability	47,260,804 (95.5)	47,236,802 (95.2)	47,330,267 (95.1)	47,496,774 (95.1)	47,650,958 (95.2)
With disability	2,231,004 (4.5)	2,372,806 (4.8)	2,432,417 (4.9)	2,435,504 (4.9)	2,415,712 (4.8)
Severity of disability among participants with disability, *n* (%)					
Severe disability	954,354 (42.8)	993,387 (41.9)	1,003,028 (41.2)	991,808 (40.7)	973,533 (40.3)
Mild disability	1,276,650 (57.2)	1,379,419 (58.1)	1,429,389 (58.8)	1,443,696 (59.3)	1,442,179 (59.7)

Characteristic	2013	2014	2015	2016	2017
All participants, *n* (%)	50,122,894	50,260,625	50,398,029	50,457,112	50,418,652
Sex, *n* (%)					
Male	25,264,937 (50.4)	25,342,530 (50.4)	25,422,698 (50.4)	25,454,109 (50.5)	25,436,486 (50.5)
Female	24,857,957 (49.6)	24,918,095 (49.6)	24,975,331 (49.6)	25,003,003 (49.6)	24,982,166 (49.6)
Age (years), mean ± SD	38.4 ± 20.7	38.8 ± 20.8	39.2 ± 20.9	39.7 ± 21.0	40.1 ± 21.1
Age group, *n* (%)					
≤19 years	10,875,214 (21.7)	10,608,339 (21.1)	10,350,132 (20.5)	10,085,711 (20.0)	9,782,482 (19.4)
20–29 years	6,784,046 (13.5)	6,846,048 (13.6)	6,909,743 (13.7)	6,967,866 (13.8)	7,003,153 (13.9)
30–39 years	8,072,634 (16.1)	7,890,347 (15.7)	7,775,698 (15.4)	7,656,016 (15.2)	7,506,124 (14.9)
40–49 years	8,707,917 (17.4)	8,720,894 (17.4)	8,647,762 (17.2)	8,575,114 (17.0)	8,476,164 (16.8)
50–59 years	7,639,506 (15.2)	7,823,118 (15.6)	7,921,992 (15.7)	8,002,761 (15.9)	8,051,108 (16.0)
60–69 years	4,125,699 (8.2)	4,331,295 (8.6)	4,657,136 (9.2)	4,920,432 (9.8)	5,165,276 (10.2)
70–79 years	2,781,383 (5.6)	2,821,653 (5.6)	2,822,542 (5.6)	2,849,701 (5.7)	2,947,195 (5.9)
≥80 years	1,136,495 (2.3)	1,218,931 (2.4)	1,313,024 (2.6)	1,399,511 (2.8)	1,487,150 (3.0)
Status of disability, *n* (%)					
Without disability	47,733,577 (95.2)	47,894,212 (95.3)	48,044,571 (95.3)	48,101,498 (95.3)	48,053,570 (95.3)
With disability	2,389,317 (4.8)	2,366,413 (4.7)	2,353,458 (4.7)	2,355,614 (4.7)	2,365,082 (4.7)
Severity of disability among participants with disability, *n* (%)					
Severe disability	956,856 (40.0)	944,316 (39.9)	935,449 (39.7)	931,343 (39.5)	928,735 (39.3)
Mild disability	1,432,461 (60.0)	1,422,097 (60.1)	1,418,009 (60.3)	1,424,271 (60.5)	1,436,347 (60.7)

Abbreviation: SD = standard deviation.

In the most recent year (2017), the number of individuals with and without disabilities was 2,365,082 (4.7%) and 48,053,570 (95.3%), respectively. The results of the comparison of the two groups are presented in Table 2. People with disability were older (59.7 ± 18.3 years in the with‐disability group and 39.2 ± 20.8 years in the without disability group, *p* < 0.001), and the proportion of men with disabilities was higher (58.1% versus 50.1%, *p* < 0.001). Among the disabled, metropolitan residents (54.7% versus 62.8%, *p* < 0.001) were lower, and the percentage of those using medical aids according to income level was significantly higher (17.0% versus 2.0%, *p* < 0.001). In people with disability, the number of individuals with comorbidities such as diabetes, chronic kidney disease, and chronic obstructive pulmonary disease (COPD) and the comorbidity scores measured by Charlson comorbidity index were also significantly higher compared with those without disability (all *p* < 0.001). People with severe disability accounted for 39.3% of the disabled, with physical disability representing the highest percentage (50.6%) (Table 2).

**Table 2:** Comparison of the General Characteristics of People With and Without Disabilities in the Most Recent Study Year (2017)

Characteristic	All participants (*n* = 50,418,652)	People without disability (*n* = 48,053,570)	People with disability (*n* = 2,365,082)	*p*
Sex, *n* (%)				<0.001
Male	25,436,486 (50.5)	24,062,915 (50.1)	1,373,571 (58.1)	
Female	24,982,166 (49.6)	23,990,655 (49.9)	991,511 (41.9)	
Age (years), mean ± SD	40.1 ± 21.1	39.2 ± 20.8	59.7 ± 18.3	<0.001
Age group, *n* (%)				
≤19 years	9,782,482 (19.4)	9,693,527 (20.2)	88,955 (3.8)	<0.001
20–29 years	7,003,153 (13.9)	6,911,756 (14.4)	91,397 (3.9)	
30–39 years	7,506,124 (14.9)	7,366,941 (15.3)	139,183 (5.9)	
40–49 years	8,476,164 (16.8)	8,201,399 (17.1)	274,765 (11.6)	
50–59 years	8,051,108 (16.0)	7,577,145 (15.8)	473,963 (20.0)	
60–69 years	5,165,276 (10.2)	4,658,937 (9.7)	506,339 (21.4)	
70–79 years	2,947,195 (5.9)	2,456,366 (5.1)	490,829 (20.8)	
≥80 years	1,487,150 (3.0)	1,187,499 (2.5)	299,651 (12.7)	
Residence, *n* (%)				<0.001
Metropolitan	31,481,734 (62.4)	30,188,762 (62.8)	1,292,972 (54.7)	
City	14,641,117 (29)	13,901,854 (28.9)	739,263 (31.3)	
Rural	4,269,197 (8.5)	3,936,363 (8.2)	332,834 (14.1)	
Unknown	26,604 (0.05)	26,591 (0.06)	13 (0.001)	
Income level, *n* (%)				<0.001
Medical aid	1,381,462 (2.7)	978,994 (2.0)	402,468 (17.0)	
First quartile	8,771,084 (17.4)	8,315,555 (17.3)	455,529 (19.3)	
Second quartile	10,052,360 (19.9)	9,692,713 (20.2)	359,647 (15.2)	
Third quartile	12,653,823 (25.1)	12,189,856 (25.4)	463,967 (19.6)	
Fourth quartile (richest)	16,385,424 (32.5)	15,734,207 (32.7)	651,217 (27.5)	
Unknown	1,174,499 (2.3)	1,142,245 (2.4)	32,254 (1.4)	
Charlson comorbidity index, *n* (%)				<0.001
0	30,009,330 (59.5)	29,221,797 (60.8)	787,533 (33.3)	
1–2	16,560,699 (32.9)	15,663,244 (32.6)	897,455 (38.0)	
3–4	2,868,479 (5.7)	2,436,061 (5.1)	432,418 (18.3)	
≥5	980,144 (1.9)	732,468 (1.5)	247,676 (10.5)	
Comorbid diseases, *n* (%)				
Diabetes mellitus				<0.001
No	47,210,434 (93.6)	45,269,028 (94.2)	1,941,406 (82.1)	
Yes	3,208,218 (6.4)	2,784,542 (5.8)	423,676 (17.9)	
Chronic kidney disease				<0.001
No	50,328,152 (99.8)	47,973,162 (99.8)	2,354,990 (99.6)	
Yes	90,500 (0.2)	80,408 (0.2)	10,092 (0.4)	
Chronic obstructive pulmonary disease				<0.001
No	44,031,722 (87.3)	42,351,962 (88.1)	1,679,760 (71.0)	
Yes	6,386,930 (12.7)	5,701,608 (11.9)	685,322 (29.0)	
Severity of disability				
Severe disability			928,735 (39.3)	
Mild disability			1,436,347 (60.7)	
Type of disability				
Physical disability			1,195,481 (50.6)	
Brain injury			232,761 (9.8)	
Facial disability			2450 (0.1)	
Visual disability			232,338 (9.8)	
Hearing disability			269,196 (11.4)	
Language disability			17,184 (0.7)	
Intellectual disability and autism			221,484 (9.4)	
Mental disability			87,830 (3.7)	
Renal disease			73,444 (3.1)	
Heart disease			6401 (0.3)	
Respiratory disease			10,679 (0.5)	
Liver disease			6041 (0.3)	
Ostomy			3041 (0.1)	
Epilepsy			6752 (0.3)	

*Note*: Value of *p* indicates a significant difference between people with and without disabilities.

Abbreviation: SD = standard deviation.

### Prevalence of osteoporosis from 2008 to 2017

Table 3 shows the crude prevalence of osteoporosis according to the disability status from 2008 to 2017. Overall, people with disabilities, including males and females, had a higher rate of osteoporosis than those without disabilities throughout the 10‐year period (Table 3, Table S2). In addition, there were significant differences in the prevalence of osteoporosis in the people with disabilities compared with those without disabilities during the same period (from 2.1% to 5.6% in people without disabilities versus from 9.4% to 21% in people with disabilities, *p* < 0.001). These differences were more pronounced in patients with mild disabilities than in those with severe disabilities (from 11.4% to 25.2% in mild disabilities versus from 6.7% to 14.6% in severe disabilities, *p* < 0.001). When assessing the prevalence of osteoporosis by year according to the type of disability, ostomy showed a steep rise, followed by hearing and physical disabilities (24%, 18%, and 13% differences, respectively). The differences of the type of disability between men and women are described in Table S2.

**Table 3:** Crude Prevalence of Osteoporosis by Disability Status, Severity, and Type from 2008 to 2017

	2008		2009		2010		2011		2012	
Parameter	Cases of OSP *n* (%)	*p*	Cases of OSP *n* (%)	*p*	Cases of OSP *n* (%)	*p*	Cases of OSP *n* (%)	*p*	Cases of OSP *n* (%)	*p*
All participants										
People without disability	1,007,835 (2.1)		1,242,516 (2.6)		1,467,125 (3.1)		1,680,159 (3.5)		1,897,165 (4.0)	
People with disability	210,451 (9.4)	<0.001	286,606 (12.1)	<0.001	339,952 (14.0)	<0.001	374,627 (15.4)	<0.001	402,060 (16.6)	<0.001
Severity of disability		<0.001		<0.001		<0.001		<0.001		<0.001
Severe disability	64,344 (6.7)		84,477 (8.5)		99,984 (10.0)		109,578 (11.0)		115,418 (11.9)	
Mild disability	146,107 (11.4)		202,129 (14.7)		239,968 (16.8)		265,049 (18.4)		286,642 (19.9)	
Type of disability		<0.001		< 0.001		<0.001		<0.001		<0.001
Physical disability	138,872 (11.4)		190,100 (14.7)		222,203 (16.8)		241,176 (18.4)		256,107 (19.8)	
Brain injury	24,002 (10.8)		31,703 (13.3)		37,107 (15.3)		41,173 (17.0)		44,772 (18.5)	
Facial disability	75 (3.5)		95 (4.2)		150 (5.6)		181 (6.5)		195 (7.1)	
Visual disability	16,421 (7.3)		21,976 (9.4)		26,783 (11.2)		30,602 (12.8)		34,059 (14.2)	
Hearing disability	21,265 (9.8)		29,894 (12.7)		37,746 (15.3)		43,315 (17.4)		47,230 (19.3)	
Language disability	395 (2.9)		497 (3.5)		644 (4.4)		784 (5.3)		899 (6.0)	
Intellectual disability and autism	1458 (0.9)		2050 (1.2)		2577 (1.4)		2977 (1.6)		3416 (1.8)	
Mental disability	1653 (2.2)		2337 (2.9)		2877 (3.4)		3270 (3.8)		3474 (4.0)	
Renal disease	2883 (5.9)		3654 (7.1)		4245 (8.0)		4871 (8.8)		5652 (9.7)	
Heart disease	1166 (7.8)		1496 (9.7)		1659 (11.4)		1636 (12.9)		1430 (14.0)	
Respiratory disease	1605 (11.1)		1958 (13.0)		2199 (15.0)		2278 (16.3)		2364 (17.8)	
Liver disease	183 (3.9)		216 (4.3)		250 (5.1)		276 (5.6)		329 (6.7)	
Ostomy	290 (9.7)		353 (12.7)		1158 (26.1)		1649 (32.1)		1650 (33.4)	
Epilepsy	183 (2.0)		277 (2.9)		354 (3.6)		439 (4.6)		483 (5.4)	

	2013		2014		2015		2016		2017	
Parameter	Cases of OSP *n* (%)	*p*	Cases of OSP *n* (%)	*p*	Cases of OSP *n* (%)	*p*	Cases of OSP *n* (%)	*p*	Cases of OSP *n* (%)	*p*
All participants										
People without disability	2,089,691 (4.4)		2,257,859 (4.7)		2,407,143 (5.0)		2,544,824 (5.3)		2,679,270 (5.6)	
People with disability	423,438 (17.7)	<0.001	440,214 (18.6)	<0.001	455,738 (19.4)	<0.001	475,284 (20.2)	<0.001	497,490 (21.0)	<0.001
Severity of disability		<0.001		<0.001		<0.001		<0.001		<0.001
Severe disability	120,072 (12.5)		123,532 (13.1)		126,677 (13.5)		130,763 (14.0)		135,187 (14.6)	
Mild disability	303,366 (21.2)		316,682 (22.3)		329,061 (23.2)		344,521 (24.2)		362,303 (25.2)	
Type of disability		<0.001		<0.001		<0.001		<0.001		<0.001
Physical disability	267,473 (21.0)		276,102 (22.0)		283,941 (23.0)		289,614 (23.8)		294,558 (24.6)	
Brain injury	47,009 (19.8)		49,059 (20.9)		51,426 (21.9)		53,286 (22.8)		54,970 (23.6)	
Facial disability	216 (8.0)		235 (8.9)		228 (9.2)		237 (9.6)		243 (9.9)	
Visual disability	36,996 (15.5)		39,259 (16.6)		41,323 (17.6)		43,083 (18.4)		44,722 (19.2)	
Hearing disability	50,317 (21.0)		52,525 (22.3)		54,707 (23.5)		62,974 (25.5)		74,902 (27.8)	
Language disability	1020 (6.8)		1138 (7.5)		1245 (7.9)		1330 (8.1)		1449 (8.4)	
Intellectual disability and autism	3908 (2.0)		4441 (2.2)		4986 (2.4)		5573 (2.6)		6182 (2.8)	
Mental disability	3799 (4.4)		4073 (4.7)		4411 (5.1)		4942 (5.6)		5338 (6.1)	
Renal disease	6481 (10.7)		7170 (11.2)		7880 (11.7)		8642 (12.3)		9469 (12.9)	
Heart disease	1299 (14.9)		1189 (15.3)		1157 (16.0)		1127 (16.6)		1100 (17.2)	
Respiratory disease	2403 (19.2)		2403 (20.4)		2397 (21.2)		2367 (21.6)		2369 (22.2)	
Liver disease	359 (7.1)		407 (7.9)		455 (8.3)		506 (8.8)		543 (9.0)	
Ostomy	1650 (35.1)		1688 (36.7)		1021 (31.9)		1025 (33.3)		1032 (33.9)	
Epilepsy	508 (6.2)		525 (7.0)		561 (7.8)		578 (8.3)		613 (9.1)	

*Note*: The value of the denominator for the corresponding disability type is placed in Table S1. Value of *p* indicates a significant difference among people without disabilities and each disability group.

Abbreviation: OSP = osteoporosis.

**Fig. 2:**
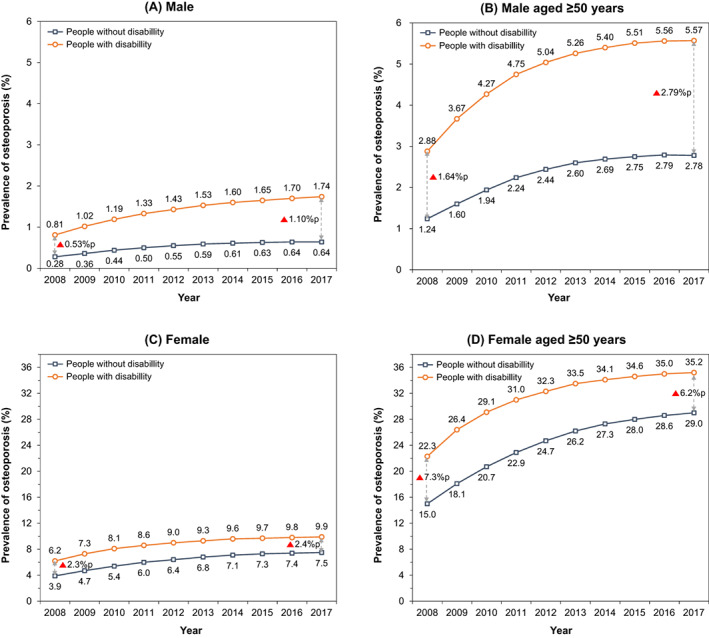
Age‐standardized prevalence of osteoporosis by disability status from 2008 to 2017. Age standardization was made using the age structure of general population in the 2005 Population and Housing Census of Korea as the standard population.

**Fig. 3:**
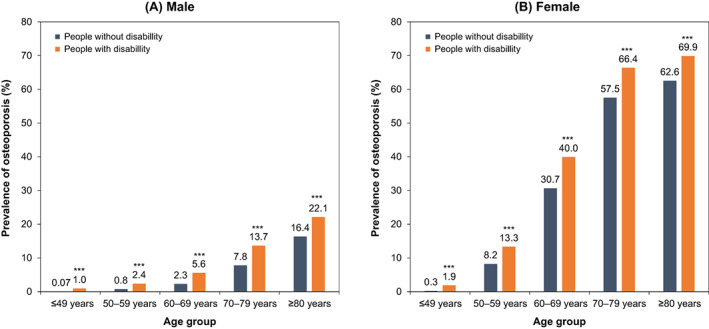
Crude prevalence of osteoporosis by age group and disability status in the most recent study year. Asterisks indicate statistically significant differences between people with and without disabilities: ***p < 0.001.

The age‐standardized prevalence of osteoporosis was also higher in both male and female individuals with disabilities than in those without disabilities in the most recent year analysis according to age group, sex, and disability status. The overall prevalence of osteoporosis was higher with age (Fig. 2A–D). The gap in the prevalence of osteoporosis according to disability status showed the greatest difference in men in their 70s and women in their 60s (Fig. 3A,B). In the age‐standardized analysis by severity and type of disability, osteoporosis in mild disability was more prevalent in women older than 50 years of age than in those with severe disability, and the most prevalent type of disability was respiratory disease in both the female and male groups (Table S3).

### Risk factors for osteoporosis

The results of the multivariate analysis by sex, disability grade, and type are presented in Table 4. People with disability had a higher risk for osteoporosis (in both sexes) than those without disability (adjusted odds ratio [aOR] 1.72; 95% confidence interval [CI] 1.70–1.73 in males; aOR 1.28, 95% CI 1.27–1.28 in women). Similar results were observed in the 50 years and older groups (aOR 1.63, 95% CI 1.62–1.65 in men; aOR 1.28; 95% CI 1.28–1.29 in women).

**Table 4:** Crude and Adjusted ORs for Osteoporosis According to Disability Characteristics in the Most Recent Year Available (2017)

	All			≥50 years		
	Crude	Age‐adjusted	Multivariate‐adjusted	Crude	Age‐adjusted	Multivariate‐adjusted
Characteristic	OR (95% CI)^a^	OR (95% CI)^b^	OR (95% CI)^c^	OR (95% CI)^a^	OR (95% CI)^b^	OR (95% CI)^c^
Male						
People without disability	1.00 (Ref.)	1.00 (Ref.)	1.00 (Ref.)	1.00 (Ref.)	1.00 (Ref.)	1.00 (Ref.)
People with disability	6.73 (6.67–6.78)	2.09 (2.07–2.10)	1.72 (1.70–1.73)	2.98 (2.96–3.01)	1.97 (1.95–1.99)	1.63 (1.62–1.65)
Severity of disability						
Severe disability	5.98 (5.90–6.05)	2.27 (2.24–2.30)	1.68 (1.66–1.70)	3.01 (2.97–3.05)	2.06 (2.03–2.09)	1.54 (1.51–1.56)
Mild disability	7.24 (7.17–7.31)	1.99 (1.97–2.01)	1.74 (1.72–1.76)	2.97 (2.94–3.00)	1.93 (1.91–1.95)	1.69 (1.67–1.71)
Type of disability						
Physical disability	6.96 (6.89–7.04)	2.44 (2.41–2.47)	2.09 (2.06–2.11)	2.86 (2.83–2.89)	2.31 (2.28–2.33)	1.97 (1.95–1.99)
Brain injury	9.90 (9.71–10.10)	2.35 (2.30–2.40)	1.73 (1.70–1.77)	3.82 (3.74–3.90)	2.19 (2.15–2.24)	1.63 (1.60–1.67)
Facial disability	2.01 (1.37–2.94)	1.38 (0.93–2.05)	1.26 (0.85–1.87)	1.06 (0.70–1.61)	1.20 (0.79–1.84)	1.10 (0.72–1.68)
Visual disability	5.68 (5.55–5.82)	1.44 (1.41–1.48)	1.29 (1.26–1.33)	2.46 (2.40–2.53)	1.41 (1.38–1.45)	1.27 (1.24–1.30)
Hearing disability	11.00 (10.80–11.20)	1.36 (1.34–1.39)	1.21 (1.18–1.23)	4.06 (3.98–4.13)	1.37 (1.34–1.40)	1.22 (1.20–1.25)
Language disability	3.67 (3.33–4.05)	1.74 (1.56–1.93)	1.35 (1.21–1.50)	2.19 (1.98–2.43)	1.63 (1.47–1.82)	1.28 (1.14–1.42)
Intellectual disability and autism	1.33 (1.27–1.39)	3.19 (3.04–3.35)	1.89 (1.80–1.99)	1.76 (1.67–1.87)	2.63 (2.48–2.79)	1.56 (1.47–1.66)
Mental disability	2.39 (2.24–2.54)	2.16 (2.02–2.30)	1.27 (1.19–1.36)	1.25 (1.17–1.34)	2.03 (1.89–2.17)	1.20 (1.12–1.29)
Renal disease	6.00 (5.75–6.26)	2.10 (2.01–2.20)	1.31 (1.25–1.37)	2.09 (2.00–2.19)	1.66 (1.59–1.75)	1.04 (0.99–1.10)
Heart disease	7.24 (6.38–8.22)	1.76 (1.54–2.01)	1.35 (1.18–1.54)	2.94 (2.58–3.36)	1.63 (1.43–1.87)	1.26 (1.10–1.45)
Respiratory disease	17.29 (16.23–18.43)	3.37 (3.16–3.61)	2.07 (1.93–2.21)	5.75 (5.39–6.13)	3.21 (3.00–3.44)	1.98 (1.85–2.12)
Liver disease	4.62 (3.96–5.40)	3.22 (2.75–3.78)	2.38 (2.03–2.80)	1.79 (1.51–2.11)	2.78 (2.35–3.29)	2.07 (1.75–2.45)
Ostomy	12.68 (10.76–14.95)	2.26 (1.89–2.70)	1.99 (1.66–2.38)	5.11 (4.31–6.07)	2.09 (1.74–2.51)	1.85 (1.54–2.22)
Epilepsy	3.45 (2.87–4.14)	3.46 (2.86–4.18)	2.16 (1.78–2.61)	2.10 (1.73–2.55)	3.32 (2.72–4.05)	2.08 (1.70–2.55)
Female						
People without disability	1.00 (Ref.)	1.00 (Ref.)	1.00 (Ref.)	1.00 (Ref.)	1.00 (Ref.)	1.00 (Ref.)
People with disability	6.32 (6.29–6.34)	1.46 (1.45–1.47)	1.28 (1.27–1.28)	2.61 (2.60–2.62)	1.46 (1.46–1.47)	1.28 (1.28–1.29)
Severity of disability						
Severe disability	3.42 (3.40–3.45)	1.08 (1.07–1.09)	0.89 (0.88–0.90)	1.76 (1.74–1.77)	1.07 (1.06–1.08)	0.88 (0.87–0.89)
Mild disability	8.86 (8.82–8.91)	1.66 (1.65–1.67)	1.49 (1.48–1.50)	3.12 (3.10–3.13)	1.67 (1.66–1.68)	1.50 (1.49–1.51)
Type of disability						
Physical disability	8.97 (8.91–9.02)	1.91 (1.89–1.92)	1.70 (1.69–1.71)	3.17 (3.16–3.19)	1.88 (1.87–1.89)	1.67 (1.66–1.68)
Brain injury	6.77 (6.68–6.85)	1.13 (1.11–1.15)	0.89 (0.88–0.90)	2.58 (2.55–2.62)	1.17 (1.15–1.19)	0.92 (0.91–0.94)
Facial disability	2.38 (2.05–2.77)	1.57 (1.31–1.88)	1.42 (1.18–1.71)	1.36 (1.15–1.60)	1.51 (1.25–1.81)	1.36 (1.13–1.63)
Visual disability	5.88 (5.81–5.96)	1.13 (1.11–1.14)	1.04 (1.03–1.06)	2.27 (2.24–2.30)	1.17 (1.15–1.19)	1.08 (1.07–1.10)
Hearing disability	8.16 (8.07–8.25)	0.92 (0.91–0.94)	0.85 (0.84–0.86)	2.92 (2.89–2.95)	1.03 (1.01–1.04)	0.95 (0.94–0.96)
Language disability	2.21 (2.07–2.37)	1.14 (1.04–1.24)	0.94 (0.86–1.03)	1.38 (1.28–1.49)	1.09 (1.00–1.19)	0.90 (0.83–0.98)
Intellectual disability and autism	0.50 (0.49–0.52)	1.03 (0.99–1.07)	0.81 (0.78–0.84)	0.60 (0.58–0.62)	0.90 (0.86–0.93)	0.69 (0.66–0.71)
Mental disability	1.00 (0.97–1.03)	0.76 (0.73–0.78)	0.64 (0.62–0.66)	0.48 (0.47–0.50)	0.68 (0.66–0.71)	0.56 (0.54–0.58)
Renal disease	2.68 (2.61–2.75)	0.86 (0.83–0.89)	0.57 (0.55–0.59)	1.01 (0.98–1.03)	0.78 (0.75–0.80)	0.51 (0.50–0.53)
Heart disease	4.65 (4.28–5.06)	1.19 (1.07–1.32)	0.93 (0.84–1.03)	2.12 (1.93–2.32)	1.17 (1.06–1.30)	0.92 (0.83–1.02)
Respiratory disease	7.19 (6.67–7.75)	2.72 (2.49–2.97)	1.74 (1.60–1.90)	2.51 (2.32–2.72)	2.40 (2.21–2.62)	1.54 (1.41–1.68)
Liver disease	1.91 (1.71–2.14)	1.41 (1.25–1.60)	1.12 (0.99–1.27)	0.87 (0.77–0.98)	1.16 (1.02–1.31)	0.91 (0.80–1.04)
Ostomy	11.17 (10.10–12.34)	1.47 (1.30–1.66)	1.31 (1.16–1.48)	4.01 (3.60–4.47)	1.50 (1.33–1.69)	1.35 (1.19–1.52)
Epilepsy	1.72 (1.56–1.90)	2.18 (1.96–2.44)	1.71 (1.53–1.91)	1.07 (0.96–1.19)	1.91 (1.70–2.14)	1.47 (1.31–1.65)

Abbreviation: CI = confidence interval; OR = odds ratio; Ref. = reference.

^a^ Not adjusted.

^b^ Adjusted for age.

^c^ Adjusted for age, residence, income level, Charlson comorbidity index, diabetes mellitus, chronic kidney disease, and chronic obstruction pulmonary disease.

The risk factors, according to the severity and type of disability, showed different results in men and women. The risk of osteoporosis was high in all subtypes of disability in the male group with disability compared with the male group without disability. The OR for osteoporosis was high in both mild and severe disability groups without a difference according to the severity of the disorder. In particular, the risk of osteoporosis was highest in people with disabilities associated with liver disease (aOR 2.38, 95% CI 2.03–2.80), epilepsy (aOR 2.16, 95% CI 1.78–2.61), respiratory disease (aOR 2.07, 95% CI 1.93–2.21), and physical disability (aOR 2.09, 95% CI 2.04–2.11). In contrast, in the female group, the risk of osteoporosis was significantly high in the mild disability group (aOR 1.49, 95% CI 1.48–1.50) according to severity, respiratory disease‐related disability (aOR 1.74, 95% CI 1.60–1.90), epilepsy (aOR 1.71, 95% CI 1.53–1.91), and physical disability (aOR 1.70, 95% CI 1.69–1.71, Table 4) related with disability type.

## Discussion

In this study, the prevalence and risk of osteoporosis were higher in people with disabilities than in those without disabilities. The prevalence of osteoporosis between the groups with and without disability showed a significant difference over time. This increase showed a marked trend in types such as ostomy, hearing, and physical disabilities.

It is known that the most dominant risk factor for osteoporosis is age.^(^ ^5^ ^)^ When the age‐standardized prevalence of osteoporosis was analyzed with the most recent data from 2017, a high percentage of disability subtypes were respiratory disease and epilepsy in both the male and female groups in our study. Previous studies have reported that the risk of osteoporosis increases in patients with respiratory diseases including chronic obstructive lung disease,^(^ ^26^, ^27^ ^)^ asthma,^(^ ^28^ ^)^ and interstitial lung disease.^(^ ^29^ ^)^ There are various causes for this; in particular, inhaled corticosteroids or systemic corticosteroids seem to act as important factors.^(^ ^28^ ^)^ The association between epilepsy, use of antiepileptic drugs, and risk of osteoporotic fractures is well recognized. Several studies have reported that patients with epilepsy have an increased risk of skeletal fractures compared with the general population.^(^ ^30^, ^31^, ^32^ ^)^

In the most recent data analysis, the top three types of disability with high multivariate‐OR for osteoporosis in both sexes were respiratory disease (OR 2.07, 95% CI 1.93–2.21 in the male group; OR 1.74, 95% CI 1.60–1.90 in the female group), epilepsy (OR 2.16, 95% CI 1.78–2.61 in the male group; OR 1.71, 95% CI 1.53–1.91 in the female group), and physical disability (OR 2.09, 95% CI 2.06–2.21 in the male group; OR 1.70, 95% CI 1.69–1.71 in the female group). A similar trend was observed in the 50 years and older group. According to the severity of disability, the mild disability group showed a tendency to have a high OR of osteoporosis compared with the severe disability group, which was prominent in women.

Several studies have examined the incidence of low BMD in both women and men with disabilities^(^ ^33^, ^34^, ^35^ ^)^ but these studies were usually physical disability‐specific, including spinal cord injury and neurologic deficits. Moreover, people who do not attain optimal bone mass during childhood and adolescence because of the early onset of disability may develop osteoporosis with accelerated bone loss.^(^ ^36^ ^)^ In addition, previous studies have reported that people with physical disabilities, such as spinal cord injuries, also had various complications of fragility fractures, delayed diagnosis due to sensory loss, pressure sores, delayed union or nonunion, hip and knee joint loss of range of motion, increased muscle spasms, and autonomic dysreflexia.^(^ ^36^, ^37^, ^38^, ^39^, ^40^ ^)^

People with disabilities have limitations of mobility, and they also have financial problems due to decreased economic activity; our research supports this. Table 2 shows that the ratio of those in the first quartile with low income and medical aid is significantly higher in people with disabilities than in those without. As access to medical facilities decreases, there are restrictions on the prevention and treatment of osteoporosis.

Our study had several limitations. We used the ICD code to define osteoporosis and could not present BMD measurements because of the limitations of medical insurance data. However, to be covered by health insurance for osteoporosis treatment, BMD tests and application of osteoporosis ICD‐codes are required. Therefore, the application of ICD codes for osteoporosis is highly reliable in Korea, although there may be some underestimation. Second, our study did not fully evaluate factors that drugs, dietary habits, smoking, and alcohol consumption can affect osteoporosis. However, this study is significant as it is the first large‐scale population‐based study to show the prevalence of osteoporosis in people with disabilities compared to people without disabilities through a long‐term follow‐up of 10 years from 2008 to 2017. In addition, various subanalyses, including age, sex, comorbidities, severity, and type, were conducted to clarify the risk factors affecting osteoporosis in disabled people. This is also a strength of the present study.

In conclusion, people with disabilities had a high prevalence and increased risk of osteoporosis compared with people without disabilities in this study. The risk of osteoporosis was significantly high in people with respiratory diseases, epilepsy, and physical disabilities. Therefore, it is important to recognize the risk of osteoporosis in people with disabilities and actively prevent and treat it.

## Author Contributions

**Ji Hyoun Kim:** Conceptualization; writing – original draft; writing – review and editing. **So Young Kim:** Conceptualization; data curation; methodology; writing – original draft; writing – review and editing. **Jong Eun Park:** Conceptualization; data curation; formal analysis; investigation; methodology; writing – review and editing. **Hyo Jong Kim:** Conceptualization; writing – review and editing. **Hyun Jeong Jeon:** Conceptualization; writing – review and editing. **Yeon Yong Kim:** Investigation; methodology; resources; writing – review and editing. **Jong‐Hyock Park:** Conceptualization; funding acquisition; project administration; supervision; writing – review and editing.

## Disclosures

All authors have completed the ICMJE uniform disclosure form at www.icmje.org/coi_disclosure.pdf and declare no support from any organization for the submitted work; these forms are available online in the Supporting Information.

### Peer Review

The peer review history for this article is available at https://www.webofscience.com/api/gateway/wos/peer-review/10.1002/jbm4.10747.

## Supporting information


**Table S1.** Type of disability among participants with disability by year (2008–2017)
**Table S2.** Crude prevalence of osteoporosis by disability type between men and women from 2008 to 2017
**Table S3.** Age‐standardized prevalence of osteoporosis by disability severity and type in the most recent study year (2017)

## Data Availability

Data is available at https://nhiss.nhis.or.kr/bd/ab/bdaba032eng.do.
