# Supplementary material for: Nationwide Trends in Osteoporosis in Koreans With Disabilities From 2008 to 2017
Source: JBMR Plus. 2023 May 3;7(6):e10747. doi: 10.1002/jbm4.10747 (PMC10241083; doi:10.1002/jbm4.10747)
Supplement: Supplementary file 1 — Table S1. Type of disability among participants with disability by year (2008–2017) Table S2. Crude prevalence of osteoporosis by disability type between men and women from 2008 to 2017 Table S3. Age‐standardized prevalence of osteoporosis by disability severity and type in the most recent study year (2017) [file JBM4-7-e10747-s001.docx]

Supplement Table 1. Type of disability among participants with disability by year (2008–2017)

|  | 2008 | 2009 | 2010 | 2011 | 2012 |
| --- | --- | --- | --- | --- | --- |
|  | n (%) | n (%) | n (%) | n (%) | n (%) |
| Type of disability among participants with disability |  |  |  |  |  |
| Physical disability | 1,214,340 (54.4) | 1,293,569 (54.5) | 1,320,871 (54.3) | 1,314,188 (54) | 1,295,617 (53.6) |
| Brain injury | 222,704 (10.0) | 238,211 (10.0) | 243,113 (10.0) | 242,878 (10.0) | 241,838 (10.0) |
| Facial disability | 2124 (0.1) | 2246 (0.1) | 2666 (0.1) | 2792 (0.1) | 2736 (0.1) |
| Visual disability | 225,771 (10.1) | 234,900 (9.9) | 239,569 (9.8) | 239,765 (9.8) | 239,298 (9.9) |
| Hearing disability | 216,470 (9.7) | 235,109 (9.9) | 246,797 (10.1) | 248,627 (10.2) | 244,918 (10.1) |
| Language disability | 13,816 (0.6) | 14,180 (0.6) | 14,554 (0.6) | 14,865 (0.6) | 14,968 (0.6) |
| Intellectual disability and autism | 165,938 (7.4) | 173,170 (7.3) | 178,376 (7.3) | 183,864 (7.5) | 189,738 (7.9) |
| Mental disability | 74,447 (3.3) | 81,945 (3.5) | 85,316 (3.5) | 86,860 (3.6) | 86,223 (3.6) |
| Renal disease | 49,268 (2.2) | 51,669 (2.2) | 52,785 (2.2) | 55,249 (2.3) | 58,030 (2.4) |
| Heart disease | 14,909 (0.7) | 15,467 (0.7) | 14,603 (0.6) | 12,727 (0.5) | 10,238 (0.4) |
| Respiratory disease | 14,514 (0.7) | 15,025 (0.6) | 14,659 (0.6) | 13,984 (0.6) | 13,292 (0.6) |
| Liver disease | 4749 (0.2) | 5006 (0.2) | 4948 (0.2) | 4935 (0.2) | 4917 (0.2) |
| Ostomy | 3004 (0.1) | 2781 (0.1) | 4442 (0.2) | 5133 (0.2) | 4934 (0.2) |
| Epilepsy | 8950 (0.4) | 9528 (0.4) | 9718 (0.4) | 9637 (0.4) | 8965 (0.4) |
| Data are presented as n (%) or mean ± standard deviation. | | | | | |

Supplemental Table 1. Continued.

|  | 2013 | 2014 | 2015 | 2016 | 2017 |
| --- | --- | --- | --- | --- | --- |
|  | n (%) | n (%) | n (%) | n (%) | n (%) |
| Type of disability among participants with disability |  |  |  |  |  |
| Physical disability | 1,273,905 (53.3) | 1,253,334 (53.0) | 1,235,866 (52.5) | 1,215,999 (51.6) | 1,195,481 (50.6) |
| Brain injury | 237,968 (10.0) | 234,647 (9.9) | 234,470 (10.0) | 233,563 (9.9) | 232,761 (9.8) |
| Facial disability | 2685 (0.1) | 2653 (0.1) | 2483 (0.1) | 2456 (0.1) | 2450 (0.1) |
| Visual disability | 237,984 (10.0) | 236,099 (10.0) | 235,381 (10.0) | 233,815 (9.9) | 232,338 (9.8) |
| Hearing disability | 239,844 (10.0) | 235,738 (10.0) | 232,369 (9.9) | 246,795 (10.5) | 269,196 (11.4) |
| Language disability | 15,106 (0.6) | 15,253 (0.6) | 15,793 (0.7) | 16,388 (0.7) | 17,184 (0.7) |
| Intellectual disability and autism | 195,693 (8.2) | 201,695 (8.5) | 208,640 (8.9) | 215,270 (9.1) | 221,484 (9.4) |
| Mental disability | 86,221 (3.6) | 86,315 (3.6) | 86,952 (3.7) | 87,504 (3.7) | 87,830 (3.7) |
| Renal disease | 60,807 (2.5) | 63,852 (2.7) | 67,160 (2.9) | 70,287 (3.0) | 73,444 (3.1) |
| Heart disease | 8717 (0.4) | 7790 (0.3) | 7242 (0.3) | 6789 (0.3) | 6401 (0.3) |
| Respiratory disease | 12,499 (0.5) | 11,774 (0.5) | 11,306 (0.5) | 10,950 (0.5) | 10,679 (0.5) |
| Liver disease | 5051 (0.2) | 5183 (0.2) | 5452 (0.2) | 5736 (0.2) | 6041 (0.3) |
| Ostomy | 4700 (0.2) | 4596 (0.2) | 3196 (0.1) | 3077 (0.1) | 3041 (0.1) |
| Epilepsy | 8137 (0.3) | 7484 (0.3) | 7148 (0.3) | 6985 (0.3) | 6752 (0.3) |
| Data are presented as n (%) or mean ± standard deviation. | | | | | |

Supplement Table 2. Crude prevalence of osteoporosis by disability type between men and women from 2008 to 2017

|  | 2008 | P value | 2009 | P value | 2010 | P value | 2011 | P value | 2012 | P value |
| --- | --- | --- | --- | --- | --- | --- | --- | --- | --- | --- |
|  | Cases of OSP (%) |  | Cases of OSP (%) |  | Cases of OSP (%) |  | Cases of OSP (%) |  | Cases of OSP (%) |  |
| **Male** |  |  |  |  |  |  |  |  |  |  |
| People without disability | 66,967 (0.3) |  | 88,838 (0.4) |  | 112,259 (0.5) |  | 134,838 (0.6) |  | 156,133 (0.7) |  |
| People with disability | 29,551 (2.2) | < 0.001 | 41,157 (2.9) | < 0.001 | 50,571 (3.6) | < 0.001 | 57,967 (4.1) | < 0.001 | 63,633 (4.5) | < 0.001 |
| Severity of disability |  | < 0.001 |  | < 0.001 |  | < 0.001 |  | < 0.001 |  | < 0.001 |
| Severe disability | 12,675 (2.2) |  | 17,140 (2.9) |  | 20,840 (3.5) |  | 23,350 (4.0) |  | 24,916 (4.3) |  |
| Mild disability | 16,876 (2.2) |  | 24,017 (3.0) |  | 29,731 (3.6) |  | 34,617 (4.2) |  | 38,717 (4.7) |  |
| Type of disability |  | < 0.001 |  | < 0.001 |  | < 0.001 |  | < 0.001 |  | < 0.001 |
| Physical disability | 17,052 (2.3) |  | 23,752 (3.1) |  | 28,867 (3.7) |  | 32,655 (4.2) |  | 35,513 (4.7) |  |
| Brain injury | 4401 (3.4) |  | 6122 (4.5) |  | 7371 (5.3) |  | 8369 (6.0) |  | 9223 (6.7) |  |
| Facial disability | 8 (0.6) |  | 11 (0.8) |  | 18 (1.1) |  | 26 (1.6) |  | 28 (1.7) |  |
| Visual disability | 2177 (1.6) |  | 3046 (2.1) |  | 3881 (2.7) |  | 4610 (3.2) |  | 5251 (3.7) |  |
| Hearing disability | 3234 (2.7) |  | 4741 (3.7) |  | 6231 (4.6) |  | 7567 (5.6) |  | 8433 (6.4) |  |
| Language disability | 91 (0.9) |  | 121 (1.2) |  | 167 (1.6) |  | 202 (1.9) |  | 232 (2.2) |  |
| Intellectual disability and autism | 348 (0.3) |  | 517 (0.5) |  | 693 (0.6) |  | 814 (0.7) |  | 925 (0.8) |  |
| Mental disability | 271 (0.7) |  | 404 (0.9) |  | 517 (1.1) |  | 578 (1.3) |  | 636 (1.4) |  |
| Renal disease | 774 (2.8) |  | 970 (3.3) |  | 1129 (3.8) |  | 1316 (4.2) |  | 1524 (4.6) |  |
| Heart disease | 174 (1.9) |  | 242 (2.6) |  | 277 (3.1) |  | 296 (3.8) |  | 266 (4.2) |  |
| Respiratory disease | 848 (7.6) |  | 1020 (8.9) |  | 1133 (10.2) |  | 1175 (11.1) |  | 1212 (12.1) |  |
| Liver disease | 86 (2.5) |  | 99 (2.7) |  | 99 (2.8) |  | 102 (3.0) |  | 119 (3.5) |  |
| Ostomy | 63 (3.5) |  | 70 (4.3) |  | 117 (5.7) |  | 164 (7.5) |  | 169 (8.0) |  |
| Epilepsy | 24 (0.5) |  | 42 (0.8) |  | 71 (1.3) |  | 93 (1.7) |  | 100 (2.0) |  |
| **Female** |  |  |  |  |  |  |  |  |  |  |
| People without disability | 940,868 (4.0) |  | 1,153,678 (4.9) |  | 1,354,866 (5.7) |  | 1,545,321 (6.5) |  | 1,741,032 (7.3) |  |
| People with disability | 180,900 (20.3) | < 0.001 | 245,449 (25.2) | < 0.001 | 289,381 (28.7) | < 0.001 | 316,660 (31.2) | < 0.001 | 338,427 (33.6) | < 0.001 |
| Severity of disability |  | < 0.001 |  | < 0.001 |  | < 0.001 |  | < 0.001 |  | < 0.001 |
| Severe disability | 51,669 (13.7) |  | 67,337 (17.0) |  | 79,144 (19.6) |  | 86,228 (21.5) |  | 90,502 (23.0) |  |
| Mild disability | 129,231 (25.2) |  | 178,112 (30.9) |  | 210,237 (34.7) |  | 230,432 (37.6) |  | 247,925 (40.4) |  |
| Type of disability |  | < 0.001 |  | < 0.001 |  | < 0.001 |  | < 0.001 |  | < 0.001 |
| Physical disability | 121,820 (25.8) |  | 166,348 (31.6) |  | 193,336 (35.5) |  | 208,521 (38.4) |  | 220594 (41.1) |  |
| Brain injury | 19,601 (20.8) |  | 25,581 (25.2) |  | 29,736 (28.6) |  | 32,804 (31.6) |  | 35549 (34.3) |  |
| Facial disability | 67 (7.6) |  | 84 (9.0) |  | 132 (12.1) |  | 155 (13.7) |  | 167 (15.1) |  |
| Visual disability | 14,244 (16.2) |  | 18,930 (20.4) |  | 22,902 (24.0) |  | 25,992 (27.0) |  | 28808 (30.0) |  |
| Hearing disability | 18,031 (18.8) |  | 25,153 (23.8) |  | 31,515 (28.1) |  | 35,748 (31.5) |  | 38797 (34.6) |  |
| Language disability | 304 (7.4) |  | 376 (8.9) |  | 477 (11.0) |  | 582 (13.1) |  | 667 (14.8) |  |
| Intellectual disability and autism | 1110 (1.8) |  | 1533 (2.4) |  | 1884 (2.8) |  | 2163 (3.1) |  | 2489 (3.5) |  |
| Mental disability | 1382 (4.0) |  | 1933 (5.0) |  | 2360 (5.9) |  | 2692 (6.6) |  | 2838 (7.0) |  |
| Renal disease | 2109 (9.8) |  | 2684 (11.9) |  | 3116 (13.6) |  | 3555 (14.8) |  | 4128 (16.6) |  |
| Heart disease | 992 (17.1) |  | 1254 (20.7) |  | 1382 (24.3) |  | 1340 (27.1) |  | 1164 (29.6) |  |
| Respiratory disease | 757 (23.0) |  | 938 (26.7) |  | 1066 (30.5) |  | 1103 (32.8) |  | 1152 (35.6) |  |
| Liver disease | 97 (7.8) |  | 117 (8.6) |  | 151 (10.5) |  | 174 (11.8) |  | 210 (13.9) |  |
| Ostomy | 227 (18.9) |  | 283 (24.6) |  | 1041 (43.7) |  | 1485 (50.6) |  | 1481 (52.6) |  |
| Epilepsy | 159 (4.0) |  | 235 (5.6) |  | 283 (6.6) |  | 346 (8.2) |  | 383 (9.7) |  |
| Abbreviation: OSP, osteoporosis.  Data are presented as n (%).  P-value indicates a significant difference among people without disabilities and each disability group. | | | | | | | | | | |

Supplement Table 2. Continued.

|  | 2013 | P-value | 2014 | P-value | 2015 | P-value | 2016 | P-value | 2017 | P-value |
| --- | --- | --- | --- | --- | --- | --- | --- | --- | --- | --- |
|  | Cases of OSP (%) |  | Cases of OSP (%) |  | Cases of OSP (%) |  | Cases of OSP (%) |  | Cases of OSP (%) |  |
| **Male** |  |  |  |  |  |  |  |  |  |  |
| People without disability | 174,519 (0.7) |  | 189,996 (0.8) |  | 204,207 (0.9) |  | 215,828 (0.9) |  | 227,272 (0.9) |  |
| People with disability | 67,975 (4.9) | < 0.001 | 71,351 (5.2) | < 0.001 | 74,843 (5.5) | < 0.001 | 78,546 (5.7) | < 0.001 | 82,799 (6.0) | < 0.001 |
| Severity of disability |  | < 0.001 |  | < 0.001 |  | < 0.001 |  | < 0.001 |  | < 0.001 |
| Severe disability | 26,108 (4.6) |  | 26,974 (4.8) |  | 27,900 (5.0) |  | 28,788 (5.2) |  | 29,813 (5.4) |  |
| Mild disability | 41,867 (5.1) |  | 44,377 (5.4) |  | 46,943 (5.8) |  | 49,758 (6.1) |  | 52,986 (6.5) |  |
| Type of disability |  | < 0.001 |  | < 0.001 |  | < 0.001 |  | < 0.001 |  | < 0.001 |
| Physical disability | 37,762 (5.1) |  | 39,459 (5.4) |  | 41,060 (5.7) |  | 42,237 (6.0) |  | 43,466 (6.2) |  |
| Brain injury | 9643 (7.1) |  | 10,099 (7.6) |  | 10,625 (8.0) |  | 11,118 (8.4) |  | 11,449 (8.6) |  |
| Facial disability | 30 (1.9) |  | 37 (2.4) |  | 31 (2.1) |  | 25 (1.7) |  | 27 (1.9) |  |
| Visual disability | 5799 (4.1) |  | 6179 (4.4) |  | 6597 (4.7) |  | 6819 (4.9) |  | 7121 (5.1) |  |
| Hearing disability | 9124 (7.0) |  | 9525 (7.5) |  | 10,109 (8.1) |  | 11,502 (8.8) |  | 13,435 (9.5) |  |
| Language disability | 279 (2.6) |  | 312 (2.9) |  | 354 (3.2) |  | 373 (3.3) |  | 406 (3.4) |  |
| Intellectual disability and autism | 1067 (0.9) |  | 1231 (1.0) |  | 1380 (1.1) |  | 1565 (1.2) |  | 1744 (1.3) |  |
| Mental disability | 687 (1.5) |  | 748 (1.7) |  | 791 (1.7) |  | 929 (2.0) |  | 1014 (2.2) |  |
| Renal disease | 1718 (4.9) |  | 1873 (5.1) |  | 2047 (5.3) |  | 2150 (5.3) |  | 2318 (5.4) |  |
| Heart disease | 254 (4.7) |  | 249 (5.2) |  | 240 (5.4) |  | 253 (6.0) |  | 256 (6.5) |  |
| Respiratory disease | 1205 (12.8) |  | 1211 (13.8) |  | 1197 (14.2) |  | 1144 (14.1) |  | 1118 (14.2) |  |
| Liver disease | 124 (3.6) |  | 145 (4.2) |  | 145 (4.0) |  | 160 (4.3) |  | 166 (4.2) |  |
| Ostomy | 182 (9.0) |  | 183 (9.3) |  | 152 (9.8) |  | 157 (10.5) |  | 160 (10.8) |  |
| Epilepsy | 101 (2.2) |  | 100 (2.4) |  | 115 (2.9) |  | 114 (2.9) |  | 119 (3.2) |  |
| **Female** |  |  |  |  |  |  |  |  |  |  |
| People without disability | 1,915,172 (8.0) |  | 2,067,863 (8.6) |  | 2,202,936 (9.2) |  | 2,328,996 (9.7) |  | 2,451,998 (10.2) |  |
| People with disability | 355,463 (35.7) | < 0.001 | 368,863 (37.3) | < 0.001 | 380,895 (38.8) | < 0.001 | 396,738 (40.3) | < 0.001 | 414,691 (41.8) | < 0.001 |
| Severity of disability |  | < 0.001 |  | < 0.001 |  | < 0.001 |  | < 0.001 |  | < 0.001 |
| Severe disability | 93,964 (24.3) |  | 96,558 (25.3) |  | 98,777 (26.1) |  | 101,975 (27.1) |  | 105,374 (28.1) |  |
| Mild disability | 261,499 (42.9) |  | 272,305 (44.9) |  | 282,118 (46.7) |  | 294,763 (48.4) |  | 309,317 (50.2) |  |
| Type of disability |  | < 0.001 |  | < 0.001 |  | < 0.001 |  | < 0.001 |  | < 0.001 |
| Physical disability | 229,711 (43.5) |  | 236,643 (45.5) |  | 242,881 (47.3) |  | 247,377 (48.9) |  | 251,092 (50.5) |  |
| Brain injury | 37,366 (36.5) |  | 38,960 (38.6) |  | 4,0801 (40.4) |  | 42,168 (42.0) |  | 43,521 (43.5) |  |
| Facial disability | 186 (17.0) |  | 198 (18.3) |  | 197 (19.2) |  | 212 (20.9) |  | 216 (21.3) |  |
| Visual disability | 31,197 (32.6) |  | 33,080 (34.8) |  | 34,726 (36.6) |  | 36,264 (38.5) |  | 37,601 (40.1) |  |
| Hearing disability | 41,193 (37.4) |  | 43,000 (39.5) |  | 44,598 (41.5) |  | 51,472 (44.6) |  | 61,467 (48.2) |  |
| Language disability | 741 (16.3) |  | 826 (17.9) |  | 891 (18.6) |  | 957 (19.2) |  | 1043 (20.1) |  |
| Intellectual disability and autism | 2841 (3.9) |  | 3210 (4.3) |  | 3606 (4.6) |  | 4008 (5.0) |  | 4438 (5.4) |  |
| Mental disability | 3112 (7.6) |  | 3325 (8.1) |  | 3620 (8.7) |  | 4013 (9.6) |  | 4324 (10.2) |  |
| Renal disease | 4763 (18.3) |  | 5297 (19.6) |  | 5833 (20.7) |  | 6492 (22.1) |  | 7151 (23.4) |  |
| Heart disease | 1045 (31.2) |  | 940 (31.2) |  | 917 (32.7) |  | 874 (33.6) |  | 844 (34.6) |  |
| Respiratory disease | 1198 (38.7) |  | 1192 (40.1) |  | 1200 (41.6) |  | 1223 (43.0) |  | 1251 (45.0) |  |
| Liver disease | 235 (14.4) |  | 262 (15.1) |  | 310 (16.6) |  | 346 (17.6) |  | 377 (17.9) |  |
| Ostomy | 1468 (54.8) |  | 1505 (57.3) |  | 869 (52.8) |  | 868 (54.8) |  | 872 (56.0) |  |
| Epilepsy | 407 (11.3) |  | 425 (12.9) |  | 446 (14.1) |  | 464 (15.0) |  | 494 (16.4) |  |
| Abbreviation: OSP, osteoporosis. Data are presented as n (%).  P-value indicates a significant difference among people without disabilities and each disability group. | | | | | | | | | | |

Supplement Table 3. Age-standardized prevalence of osteoporosis by disability severity and type in the most recent study year (2017)

|  | **Male** | |  | **Female** | |
| --- | --- | --- | --- | --- | --- |
|  | All, % | ≥50 years, % |  | All, % | ≥50 years, % |
| People without disability | 0.64 | 2.78 |  | 7.5 | 29.0 |
| Severity of disability |  |  |  |  |  |
| Severe disability | 1.99 | 6.02 |  | 8.8 | 31.0 |
| Mild disability | 1.54 | 5.33 |  | 10.4 | 37.0 |
| Type of disability |  |  |  |  |  |
| Physical disability | 2.34 | 5.96 |  | 11.4 | 38.4 |
| Brain injury | 2.96 | 6.95 |  | 10.6 | 34.0 |
| Facial disability | 0.92 | 3.35 |  | 8.8 | 34.5 |
| Visual disability | 1.07 | 3.99 |  | 8.5 | 32.0 |
| Hearing disability | 1.01 | 4.17 |  | 8.5 | 32.2 |
| Language disability | 1.36 | 4.85 |  | 8.4 | 30.3 |
| Intellectual disability and autism | 1.71 | 6.27 |  | 7.2 | 26.1 |
| Mental disability | 1.39 | 5.17 |  | 5.9 | 22.4 |
| Renal disease | 4.59 | 5.35 |  | 9.4 | 24.9 |
| Heart disease | 2.08 | 5.13 |  | 9.7 | 32.5 |
| Respiratory disease | 8.21 | 9.82 |  | 14.9 | 44.0 |
| Liver disease | 2.68 | 6.19 |  | 9.9 | 30.2 |
| Ostomy | 3.62 | 6.99 |  | 15.6 | 39.7 |
| Epilepsy | 1.86 | 7.92 |  | 10.7 | 39.2 |
| Age standardization was made using the age structure of the general population in the 2005 Population and Housing Census of Korea as the standard population. | | | | | |
